# A Degradable Bioinspired Flier with Aerogel‐Based Colorimetric Sensors for Environmental Monitoring

**DOI:** 10.1002/advs.202508949

**Published:** 2025-08-28

**Authors:** Gianpaolo Gallo, Ruowen Tu, Carlo Filippeschi, Stefano Mariani, Barbara Mazzolai

**Affiliations:** ^1^ Bioinspired Soft Robotics Laboratory Istituto Italiano di Tecnologia Via Morego 30 Genova 16163 Italy

**Keywords:** aerogel, ammonia sensor, bioinspiration, colorimetric sensor, degradable flier, environmental monitoring, pH sensor

## Abstract

Environmental and distributed monitoring of remote, inaccessible, or polluted areas requires low‐maintenance and sustainable solutions. Passive dispersal strategies with (bio)degradable fliers, inspired by plant anemochory, offer an eco‐friendly approach to deploy distributed sensors with minimal human intervention. In this work, a degradable flier, inspired by *Tipuana tipu* samaras, is presented, integrating 3D printed porous cellulose nanocrystal aerogel (CNCa) sensors onto poly(vinyl alcohol) (PVA) wings. The morphology and flight behavior of natural *Tipuana tipu* samaras are characterized to guide the design and fabrication of the artificial samaras. The fliers resemble the morphometry and aerodynamic performance of natural counterparts. The CNCa sensors provide low mass, high surface area, and fast analyte diffusion, supporting large (≈3 cm^2^) readable surfaces for remote image‐based detection. Natural, edible halochromic dyes — red cabbage anthocyanins and turmeric curcumin — are embedded into CNCa for colorimetric detection of pH and gaseous ammonia level, relevant for monitoring acid rain and fertilizer emissions. The water‐soluble PVA wing promotes rapid degradation after deployment, while the aerogel sensors persist longer, supporting a two‐phase degradation strategy that balances environmental sustainability with functional longevity. The work highlights the potential of bioinspired, degradable, colorimetric fliers for in situ environmental monitoring with prospective application in precision agriculture.

## Graphical abstract

Biodegradable fliers are developed inspired by *Tipuana tipu* samaras, integrating cellulose nanocrystal aerogel (CNCa) sensors loaded with natural dyes for pH and ammonia detection. The lightweight, degradable fliers mimic natural morphology and aerodynamics, offering an eco‐friendly, scalable solution for in situ environmental monitoring after passive dispersal, with easy visual readout and a two‐step degradation process for sustainability.

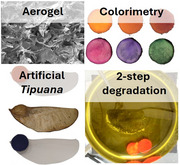

## 1 Introduction

Environmental monitoring often requires great efforts in terms of cost, maintenance, energy‐ and time‐consumption.^[^ ^1^, ^2^, ^3^, ^4^, ^5^ ^]^ In addition to the large‐scale regional monitoring enabled by satellite remote sensing, distributed sensors have recently gained popularity for providing high‐resolution monitoring in both temporal and spatial domains.^[^ ^6^, ^7^, ^8^ ^]^ For this kind of sensors, passive dispersal strategies employing external agents like wind are particularly desirable to lower the sensor distribution complexity, cost, and human intervention.^[^ ^9^, ^10^, ^11^ ^]^

Many of these passive dispersion methods are not novel; they have long been used by plants to ensure the survival and propagation of their species.^[^ ^12^ ^]^ Anemochory, or wind‐driven dispersal, is highly versatile in nature and includes mechanisms that exploit aerodynamic principles to slow descent and maximize dispersal.^[^ ^13^, ^14^, ^15^ ^]^ Maple (*Acer* genus) seeds, for example, create leading‐edge vortices during autorotation;^[^ ^14^, ^16^ ^]^ dandelions (*Taraxacum officinale*) use hairy parachute‐like structures for drag;^[^ ^17^, ^18^ ^]^ and *Alsomitra macrocarpa* employs thin airfoil‐like wings for lift.^[^ ^19^ ^]^ These dispersal mechanisms have inspired the development of artificial fliers for environmental monitoring.^[^ ^9^, ^11^, ^20^, ^21^, ^22^ ^]^

Many seed‐inspired fliers integrate electronics and non‐biodegradable materials,^[^ ^10^, ^23^, ^24^, ^25^, ^26^ ^]^ raising concerns about sustainability and e‐waste, and as a result, more eco‐friendly and (bio)degradable alternatives have recently emerged.^[^ ^9^, ^11^, ^20^, ^21^, ^22^ ^]^ Among them, flier integrating colorimetric sensors are commonly used due to their simplicity, low cost, and ease of visual detection,^[^ ^27^, ^28^ ^]^ and typically, the dye is embedded in a planar support tailored to the flier's dimensions.^[^ ^10^, ^21^, ^22^, ^29^ ^]^ However, to minimize mass for wider dispersal, flier sizes are usually under 5 cm and less than 1 g in weight, limiting sensor readout areas to ≤1 cm^2^ and making image‐based remote detection by aircraft or drones challenging.

To address this, we drew inspiration from large samara seeds, such as those of *Tipuana tipu (T. tipu)*, which exhibit characteristic autorotational descent similar to other samaras (e.g., *Acer* spp. and *Fraxinus* spp.), but possess notably larger wings and pericarps, facilitating visual tracking.^[^ ^30^ ^]^ In addition, to improve the dispersal of bioinspired artificial samara‐like sensors by reducing their mass without compromising environmental sensitivity, ultra‐high‐porosity materials such as aerogels present a promising design strategy.^[^ ^31^, ^32^ ^]^

Aerogels are porous solids created by replacing the solvent in a gel with air^[^ ^31^ ^]^ featuring low density and large surface area,^[^ ^32^ ^]^ ideal properties for environmental sensing. The porous nature enhances responsiveness and shortens response times compared to bulk or film‐like materials.^[^ ^33^, ^34^, ^35^ ^]^ Additionally, aerogels can be made from biodegradable, non‐toxic substances like cellulose derivatives, chitosan, and gelatin,^[^ ^36^ ^]^ another desired property for disposable and transient environmental devices.^[^ ^37^ ^]^ An aerogel sensor made from cellulose derivatives provides a hydrophilic support^[^ ^38^ ^]^ that can take up a large amount of water without dissolving.^[^ ^39^ ^]^ These properties, for example, have allowed cellulose‐based aerogels to be used as membranes for water purification.^[^ ^40^ ^]^ Due to their high surface area, aerogels can adsorb responsive dyes for colorimetric sensing.^[^ ^38^ ^]^ To promote safety and sustainability, natural dyes, like those from red cabbage and turmeric, are preferred over synthetic ones.^[^ ^41^, ^42^ ^]^

Red cabbage is rich in anthocyanins,^[^ ^43^, ^44^ ^]^ halochromic compounds previously used in deployable sensors for rain and topsoil pH monitoring.^[^ ^21^ ^]^ These anthocyanins could benefit from hydrophilic aerogel support, which absorbs water without dissolving.^[^ ^39^ ^]^ On the other hand, turmeric contains curcumin, a yellow dye that turns red under alkaline conditions and that can be used to detect ammonia (NH_3_).^[^ ^45^ ^]^ NH_3_ is a byproduct of fertilizer decomposition,^[^ ^46^, ^47^, ^48^ ^]^ and it is both a pollutant and a precursor of PM2.5 particles.^[^ ^47^ ^]^ For this reason, monitoring NH_3_ emissions from soil is essential for safety and for optimizing fertilizer use.^[^ ^46^, ^47^, ^48^ ^]^ The high surface area of aerogels could make them particularly effective for detecting gaseous analytes.^[^ ^32^, ^33^ ^]^

Sustainability also requires all flier components to be biodegradable and to degrade within a controlled timeframe.^[^ ^49^ ^]^ Following deployment, the flier wing becomes functionally obsolete—or potentially problematic, as in the case of unintended secondary dispersion—making its rapid degradation desirable. In contrast, the sensors used for environmental monitoring may require extended functional lifespans, thus necessitating the use of more durable yet still biodegradable materials.

In response to all these needs, we propose a *T. tipu* samara‐inspired flier incorporating cellulose nanocrystal (CNC) aerogel‐based sensors functionalized with anthocyanins and curcumin for monitoring rain pH and atmospheric NH_3_, respectively. The wing is fabricated from water‐soluble, biodegradable poly(vinyl alcohol) (PVA), allowing it to rapidly dissolve upon rainfall (e.g., less than 1 h).^[^ ^50^ ^]^ In contrast, the aerogel‐based sensors remain intact for extended environmental monitoring.^[^ ^39^, ^40^ ^]^ The system enables visible colorimetric responses to acid rain and NH_3_ over a large sensing area (≈3 cm^2^), facilitating, in perspective, remote detection through image‐based analysis, for example, using unmanned aerial vehicles (UAVs, or drones).

In summary, our work introduces, for the first time, a large‐area (≈3 cm^2^), lightweight, and highly porous (bio)degradable colorimetric sensor, based on a cellulose nanocrystal aerogel embedded with edible pH and NH_3_ indicators, designed for integration with seed‐inspired fliers. The enlarged sensing area enables more practical remote visual readout, overcoming the limitations of previous miniaturized optical sensors while maintaining favorable flight performance. In addition, the use of sustainable and biodegradable materials enhances environmental compatibility, representing a significant advancement toward practical and eco‐friendly bioinspired solutions for colorimetric and distributed environmental monitoring, and prospective application in precision agriculture.

## 2 Results

### 2.1 Colorimetric Sensors Fabrication

**Figure 1:**
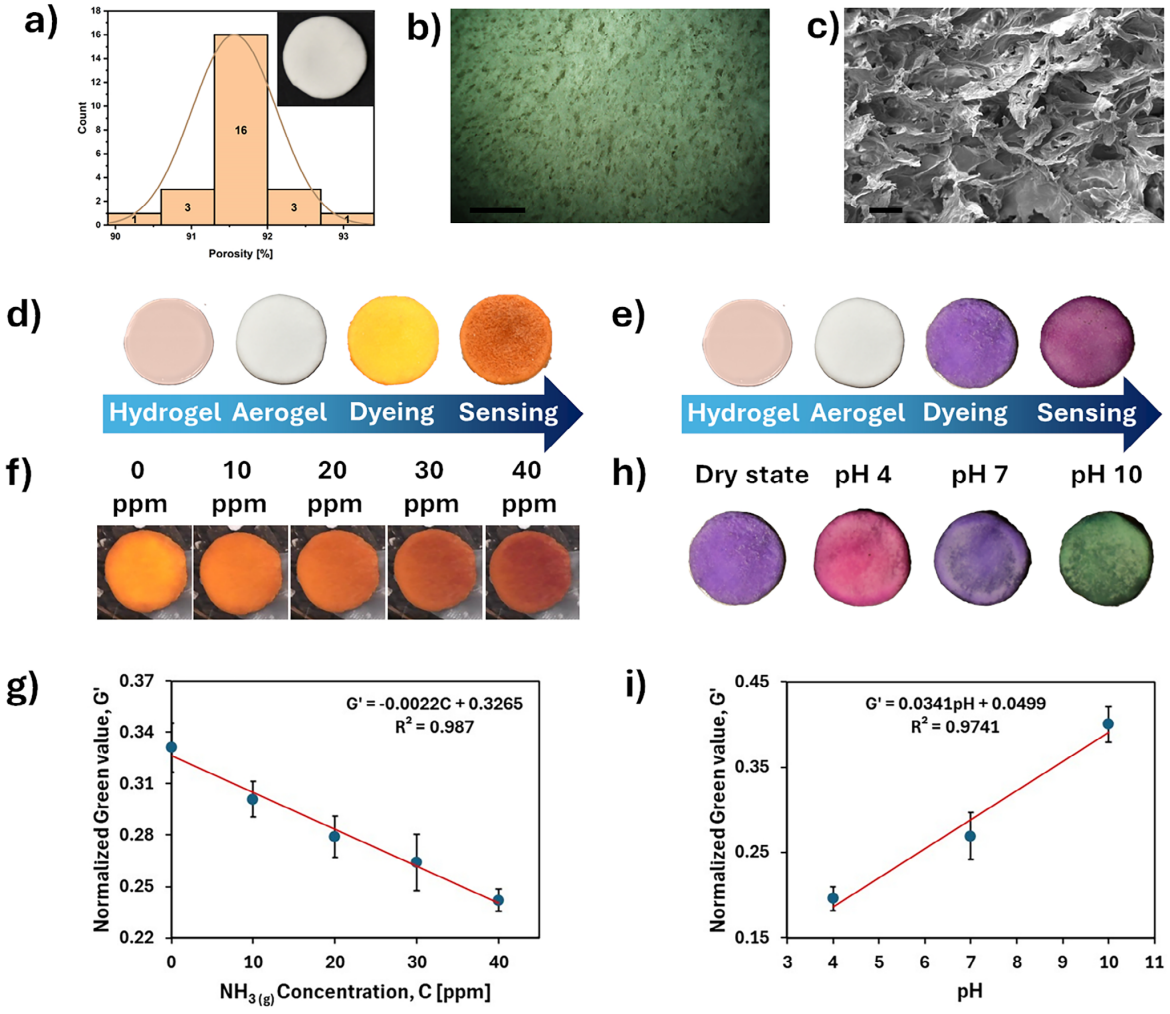
Aerogel‐based sensors. a) Aerogel's porosity distribution over 24 disks, inset shows the macroscopic appearance of one of the disks. b) Aerogel porous structure as visible from confocal microscopy, scalebar is 1000 µm. c) SEM imaging of an aerogel disk surface, scalebar is 100 µm. d) NH_3_‐ and e) pH‐responsive aerogel disks sensors fabrication steps. f) Chromatic evolution of the turmeric sensor with increasing ammonia concentration (ppm). g) Calibration curve for NH_3(g)_ sensors. h) Chromatic response of red cabbage‐based sensors before (dry state) and after the addition of different pH buffers. i) Calibration curve for pH sensors. Data are presented as mean ± SD, and error bars indicate standard deviations (N samples = 3–5).

One of the pivotal sections of the artificial *T. tipu* samara is the sensors, which adopt CNC aerogel (CNCa) disks as support for halochromic natural dyes (**Figure** **1**). The aerogel's inherent porosity facilitates the exchange between the disk structure and external species, especially in the gaseous (ammonia) state.^[^ ^51^ ^]^ The process to fabricate CNCa disks first involves the production of a CNC hydrogel (10 wt.% in deionized water), which is 3D printed into a disk shape (diameter: 20 mm, thickness 2.4 mm), using a direct ink writing (DIW) system based on pneumatic extrusion bioprinting.^[^ ^52^ ^]^

The printability of CNC hydrogels was already demonstrated in the literature,^[^ ^53^ ^]^ and are based on the properties of these hydrophilic, biodegradable nanoparticles derived from natural sources such as wood biomass.^[^ ^54^ ^]^ The CNC concentration in the hydrogel was chosen as a compromise between the rheological requirements for DIW 3D printing and the corresponding aerogel final density. The aerogel disks were fabricated using a mild‐condition process through freezing (−20 °C), thawing in ethanol, and solvent exchange with acetone, without the need for a freeze‐drier, which was adapted from the literature.^[^ ^55^ ^]^ Finally, upon ambient‐pressure evaporation, the highly volatile solvent was eliminated, yielding a porous aerogel structure.

The average mass (*m*), density (*ρ*), and porosity (*P*) of the resulting CNCa disks were measured to be *m* = 0.121 ± 0.016 g, *ρ* = 0.126 ± 0.008 g cm^−3^ and *P* = 91.6% ± 0.6%, respectively (*n* samples = 24), by considering *ρ*_CNC_ = 1.49 g cm^−3^ as the CNC density, obtained from the provider.

The CNCa porosity follows a Gaussian distribution, as shown in Figure 1a. The porous nature of the aerogel was confirmed by both optical and scanning electron microscopy (SEM) investigations (Figure 1b,c). Pores ranging from tens to hundreds of micrometers originated from ice crystal formation during freezing, while the CNC formed a physically linked, continuous network that maintained the structural integrity.

The sensing abilities of the sensors are endowed by halochromic molecules (anthocyanins and curcumin), which are present in extracts of edible, non‐toxic, vegetable species: red cabbage leaves and turmeric powder (Figure S1, Supporting Information). These extracts were used to dye the aerogel structure, whose high exchange surface allows rapid dye adsorption (Figure S2, Supporting Information). In this work, we limited our scope to turmeric and red cabbage extracts. However, dyeing of these aerogel scaffolds should be easily adaptable to any type of non‐aqueous solution containing spectroscopically active species, such as chromophore or fluorophores. The most significant fabrication steps for aerogel‐based sensors dyed with turmeric and red cabbage extracts are shown in Figure 1d,e, respectively.

For the turmeric‐based sensors, calibration was performed in a confined environment, where the simultaneous response of our colorimetric sensor and electronic reference instrument was recorded and compared, both exposed to increasing levels of NH_3(g)_ (Figure S3, Supporting Information). A 2.5 wt.% NH_4_OH aqueous solution was used as a source of gaseous ammonia. As the NH_3_ concentration (*C*, in ppm) increased, the sensor color progressively shifted from yellow to orange, with a red component becoming increasingly visible (Figure 1f). A calibration curve was obtained from RGB analysis (see Experimental Section) of video frames at different ppm levels. Particularly, the normalized green value *G′*, calculated as:

(1) $$G^{\prime }=\frac{G}{R+G+B}$$

showed a linear correlation with NH_3(g)_ concentration *C* (in ppm), with R^2^ = 0.987 (Figure 1g). The data of the calibration curve showed a good linearity and a good repeatability with an average relative standard deviation (%RSD of 5.85% ± 3.08%, N samples = 5). For anthocyanin‐based sensors, buffer solutions of pH = 4, 7, and 10 were applied drop‐by‐drop over an equal number of sensors. As expected, this caused the sensors to shift from a deep blue/violet color to magenta (pH = 4), blue (pH = 7), and green (pH = 10), respectively (Figure 1h). A similar procedure to the one described for the turmeric‐based sensor was followed to build calibration curves from RGB analysis of the sensors' color. The *G’* response showed R^2^ = 0.974 and was chosen for color‐to‐pH conversion (Figure 1i). The data of the calibration curve showed a good linearity and a good repeatability with an average relative standard deviation (%RSD of 7.55% ± 2.49%, N samples = 3).

### 2.2 Characterization of Natural *T. tipu* Samaras

**Figure 2:**
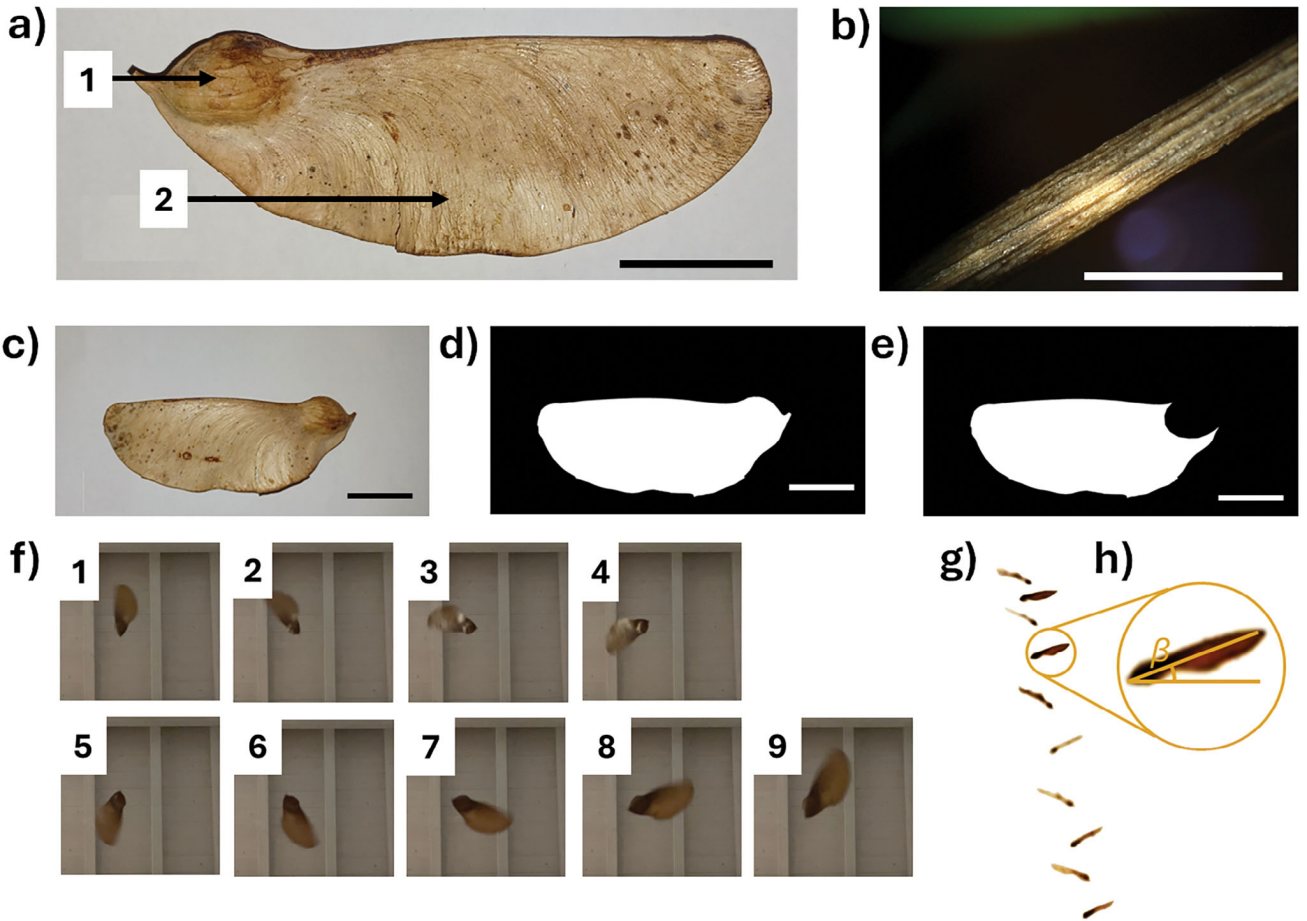
Morphometric analysis of natural *T. tipu* samara. a) Picture of *T. tipu* samara pericarp (1) and wing (2) portion. Scalebar is 2 cm. b) Picture of the major wing thickness chord. Scalebar is 1 mm. c) Picture of the *T. tipu* samara for the estimation of the wing surface. d) Image binarization of the image reported in picture c) for the wing surface (*S_w_*) estimation. e) Image binarization of the image reported in picture c) for the wing surface estimation (without capsule). In c–e) the scalebar is 2 cm. f) 9 bottom view frames sequentially captured (every 12.5 ms) showing the autorotation of a *T. tipu* samara during the falling from 3 m. g) 10 lateral view frames captured during the autorotation of a *T. tipu* samara falling from 3 m for the estimation of the coning angle *β*. c) Zoom from h) for the estimation of *β*. Frames for (f) and (g) have been captured from Videos S1 and S2.

Despite belonging to the *Fabaceae* family, the samaras of *T. tipu* are morphologically similar to other autorotating samaras, like those produced by the *Acer* genus (*Sapindaceae* family). Therefore, they possess an asymmetric, winged shape, which can be divided into the pericarp (the seed portion of a samara) in the distal position, and the thin, membranous wing, which occupies most of the samara surface (**Figure** **2**a; Figure S4a, Supporting Information). They also present an upper rib stemming from the pericarp and shaping the wing profile with an average thickness of 260 ± 14 µm (Figure 2b; Figure S4b, Supporting Information). However, compared to *Acer platanoides* the latter has a greater mass (*m* = 0.9 ± 0.2 g) and wing area (*S_w_* = 17.2 ± 1.7 cm^2^). Most of the mass of the samara is concentrated in the pericarp region, with a capsule‐to‐wing mass ratio (*m_c_/m_w_*) of 3.9 ± 0.8.

The lengths measured for morphometric analysis are shown in Figure S4 (Supporting Information), while the corresponding average values are collected in Table S1 (Supporting Information). The total surface (*S_t_*) and wing surface (*S_w_*) were calculated from binarization of pictures of the seeds (Figure 2c–e) and resulted in the values of 19.1 ± 1.7 and 17.2 ± 1.7 cm^2^, respectively. Considering *g* = 9.81 m s^−2^, this led to an average wing loading *W/S_w_* of 4.9 ± 0.7 N m^−2^, one of the highest among anemochoric species.^[^ ^13^, ^16^, ^56^ ^]^

The flight performance of selected samaras of *T. tipu* was later investigated from recordings of their mid‐air rotating behavior (Figure 2f–h; Videos S1 and S2, Supporting Information). We found an average descent velocity, *v_d_* = 1.4 ± 0.2 m s^−1^, higher than the reported value for *Acer platanoides* (lab descent speed of 1.10 ± 0.24 m s^−1^),^[^ ^57^ ^]^ and an angular velocity of *Ω* = 57.9 ± 8.3 rad s^−1^ (Figure 2f). An average conic angle *β* = 26.3 ± 3.8 ° (Figure 2g,h) was measured, a value consistent with those reported for other autorotating species.^[^ ^16^ ^]^ To calculate the wing tip speed *v_t_*, the descent factor *DF* (also known as drag coefficient, *C*_*D*_), and the Reynolds number *Re* we applied formulas found in previous literature:^[^ ^9^, ^14^, ^24^ ^]^

(2) $$v_{t}=\Omega \times r$$

(3) $$Re=\frac{v_{d}\times w\times \rho_{air}}{\mu_{air}}$$

(4) $$DF=C_{D}=\frac{2W/S_{w}}{\rho_{air}\times v_{d}^{2}}$$

where *r* is the whole samara wingspan (Table S1, Supporting Information, *b* entry), *w* the wing chord (Table S1, Supporting Information, *g* entry), *ρ_air_* is air density (1.204 kg m^−3^) and *µ_air_* the air dynamic viscosity (1.81 × 10^−5 ^kg m^−1^ s^−1^). All the aerodynamic parameters are collected in Table S2 (Supporting Information). All morphometric and aerodynamic parameters of the natural seeds were then used to design artificial seeds.

### 2.3 Artificial *T. tipu* Samaras

To design the flier, we first extracted a vectorial image of the *T. tipu* samara shape from a picture, which was then imported into CAD software to generate a 3D model (Figure S5, Supporting Information). The latter comprises an 81.3 mm‐long wing and a chord of 31 mm. The samara shape was adapted to encase a 1 mm‐thick, circular area that was later used as a base for the aerogel sensors. The radius of such an area was 10 mm, and the center of the circle corresponded to the position of the geometrical center of the seed capsule in natural *T. tipu* samaras. As part of the final model, 3, 0.6 mm‐wide and 0.3 mm‐thick ribs were also included on top of the 0.1 mm‐thick wing area. These served to increase wing rigidity and ensured better aerodynamic performance during the flight, while keeping the wing mass at a minimum. The outermost rib follows the curved outline of the natural upper rib of *T. tipu* samaras, while the other two are linear and span the wing area. We chose a PVA filament as the printing material for fused deposition modelling (FDM) 3D printing, since it is soluble in water, non‐toxic, and biodegradable.^[^ ^58^ ^]^ The final flier had a wing area *S_w_* = 15.9 ± 0.5 cm^2^, slightly lower than the natural one (i.e, 17.2 ± 1.7 cm^2^). However, the pericarpal area was increased to make space for 3D aerogel‐based sensors of an area close to 3 cm^2^.

**Figure 3:**
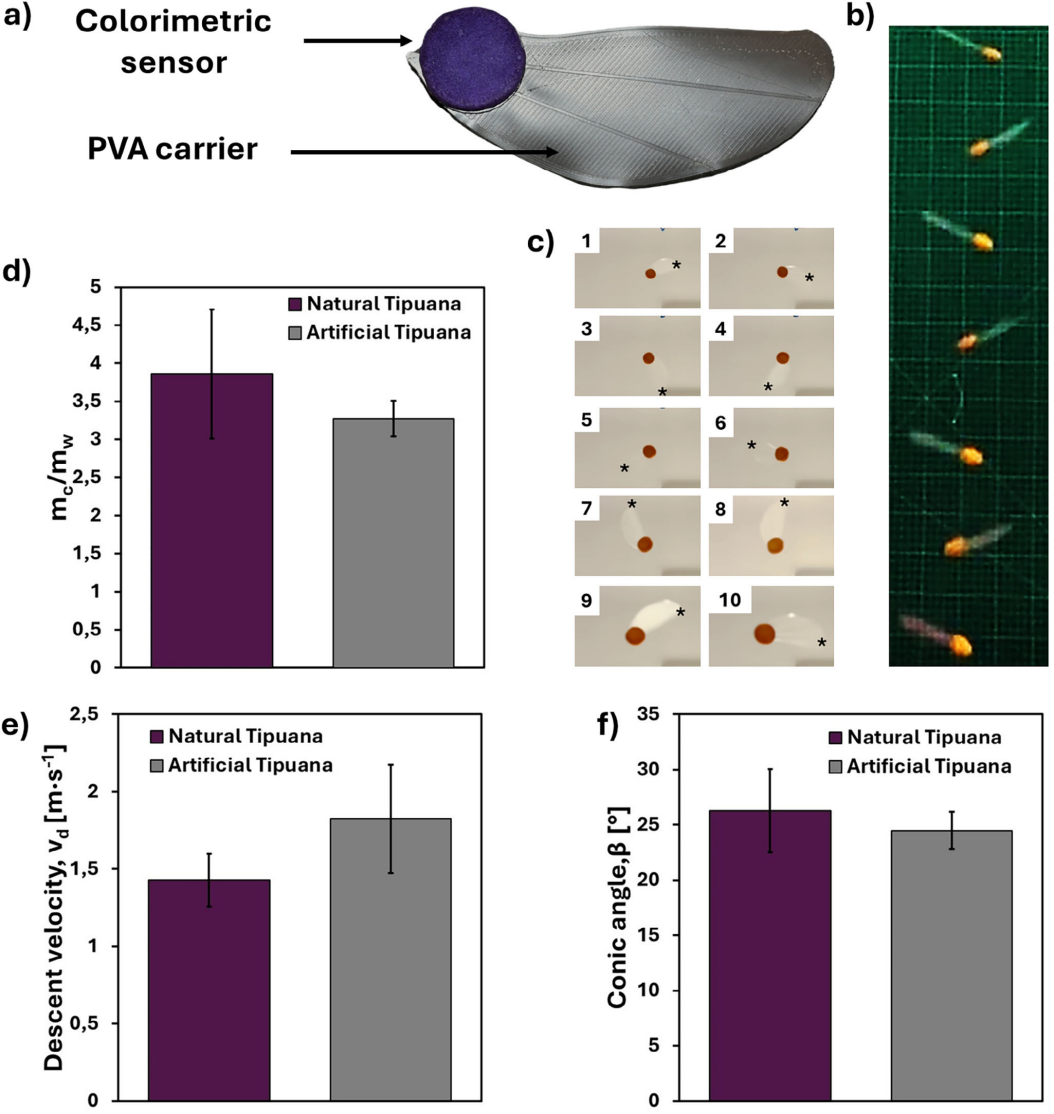
Aerodynamic analysis of the natural and artificial *T. tipu* samara. a) Picture of the artificial *T. tipu* samara and its two components. b) 13 lateral view frames captured and overlayed (Video S3, Supporting Information) during the autorotation of an artificial *T. tipu* samara falling from 3 m for the estimation of the conic angle (*β*). c) 10 bottom view frames sequentially captured (every ≈12 ms, see Video S4, Supporting Information) showing the autorotation of an artificial *T. tipu* samara during the falling from 3 m. d–f) Comparison between the natural and artificial *T. tipu* samaras’ parameters: d) *m_c_/m_w_* ratio, e) descent velocity, *v_d_*, f) conic angle *β*. Data are presented as mean ± SD, and error bars indicate standard deviations (N samples = 3–6).

**Figure** **3**a shows the fully assembled artificial *T. tipu* samara incorporating a colorimetric CNCa sensors. The application of sensors on both sides of the artificial samara ensures that at least one sensor is always readily accessible and readable from above, regardless of the landing side of the samara. Moreover, it helped to reach an *m_c_/m_w_* ratio of 3.3 ± 0.2 and a whole samara mass of *m* = 0.91 ± 0.04 g. These values are not significantly different (Student's *t*‐test, α = 0.05) from the obtained from their natural counterparts (*m* = 0.9 ± 0.2 g, *m_c_/m_w_* = 3.9 ± 0.8). The same is true for their wing loading *W/S_w_*, 5.08 ± 0.56 and 5.59 ± 0.30 N m^−2^ for natural and artificial counterpart, respectively. The flying behavior of the artificial samaras was studied as already described for the natural samaras (Figure 3b,c; Videos S3 and S4, Supporting Information), and a comparison between the two cases is reported in Table S3 (Supporting Information). The descent speed of the artificial samara, *v_d_* = 1.8 ± 0.3 m s^−1^ was significantly higher (Student's *t*‐test, α = 0.05) compared to the natural one (1.4 ± 0.2 m s^−1^). However, if considering a confidence level of α = 0.01 we could reject the null hypothesis, meaning that the ability of the artificial samara to slow down its descent was not significantly different from the natural one. Another important difference was encountered by looking at the rotational velocity (*Ω =* 57.9 ± 8.3 rad s^−1^) and wing tip speed (*v_t_ =* 4.4 ± 0.7 m s^−1^) of the artificial samara, which were lower than in the natural case (*Ω =* 67.6 ± 3.2 rad s^−1^, *v_t_* = 5.5 ± 0.3, s^−1^), despite the former had a higher wingspan (*b* = 81.5 ± 0.2 mm, compared to 76 ± 4 mm for the natural case). With an average *β =* 26.3 ± 3.8 °, the conic angle of natural *T. tipu* samples was comparable to their manufactured counterparts *β* = 24.2 ± 1.7 °. The same is true for *Re* and *DF*, of comparable magnitude between the two cases (Figure S6, Supporting Information). Graphical comparisons between the natural (purple) and artificial (gray) samara *m_c_/m_w_*, *v_d,_* and *β* values are shown in Figure 3d–f, while all the other parameters are collected and compared in Figure S6 (Supporting Information).

In the future, the samara design could be further improved to carefully reduce its wing loading. The wing thickness was already reduced to a minimum (≈0.10 mm) for the current FDM printer and instrumentation used. The sensor base could be further thinned out, but this would influence the *m*_c_/*m*_w_ and, indirectly, its flight performance. Despite this, we developed a simple and versatile fabrication process for the whole samara, which overall led to comparable performances to the natural case.

### 2.4 Sensing and Degradation in Simulated Environments

**Figure 4:**
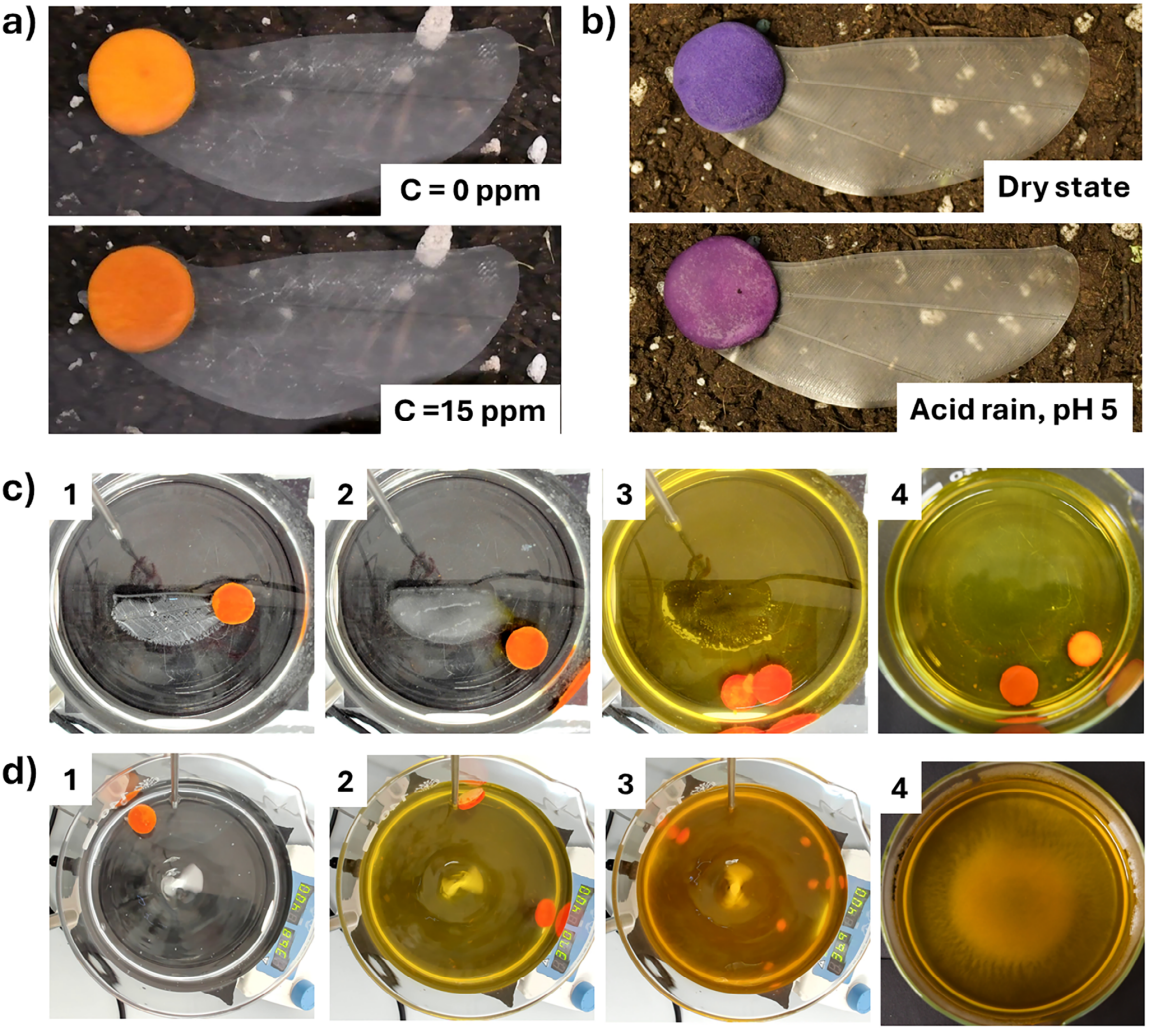
Artificial *T. tipu* samara behavior in simulated conditions. a) NH_3_‐sensitive samara before and after exposure to a gaseous ammonia level *C* = 15 ppm; b) pH‐sensitive samara before and after exposure to simulated acid rain (pH = 5 acetate buffer); Accelerated degradation experiments on artificial *T. tipu* samaras in PBS (500 mL, pH = 7.40) at 37 °C in c) static (0 rpm, revolutions per minute) conditions after 0 s, ≈7 min, 3 h, 18 h or d) dynamic conditions (400 rpm) after 0 s, 20 min, 3 h, 5 h.

To demonstrate the use of our artificial *T. tipu* samara in real‐world scenarios, we tested its behavior under different simulated conditions. We started by evaluating their sensing abilities. Nitrogen loss from fertilized areas depends on many factors, such as soil pH, oxygen levels, type of fertilizer.^[^ ^59^, ^60^ ^]^ Previous research studies have shown, for example, that spikes as high as 150 ppm of gaseous ammonia are observed right after manure application on topsoil. However, levels quickly decay over time and, after hours, values below 40 ppm are observed at the topsoil/air interface.^[^ ^59^ ^]^ The latter value is in line with the linear response range of 0–40 ppm analyzed in Section 2.1. Considering the time required for both fertilizer and sensor distribution over wide areas, we envision our artificial *T. tipu* samara to be suitable for quick and quantitative assessment of ammonia evolution within the latter timeframe, where ammonia emission seems to be regulated by complex soil pH, oxygen, and microbial dynamics.^[^ ^59^ ^]^ To demonstrate the accuracy of the calibration curve (Figure 1g) we tested a chromatic response (**Figure** **4**a), in terms of *G’*, of three fliers coupled to ammonia sensors exposed to a *C* = 15 ppm. The same setup and conditions described for the calibration data were employed. The observed average ammonia concentration *C* = 16 ± 4 ppm (N samples = 3) obtained from this analysis showed our sensors had a 96% accuracy (Figure S7a, Supporting Information), confirming the reliability of the linear calibration reported in Figure 1g. The response time of the NH_3_ sensors for a concentration of 15 ppm (expressed in seconds) was 189 ±60 s (N samples = 5). A similar procedure was repeated on three pH‐sensitive artificial samaras. The latter were exposed to an acidic buffer (pH = 5), simulating an acid rain event. The sensors responded by turning to a purple color (Figure 4b), indicating an acidic environment. The observed mean pH value was 5.2 ± 0.2 (N samples = 3), representing an accuracy of 95% (Figure S7b, Supporting Information), confirming the reliability of the linear calibration reported in Figure 1i. In the case of the simulated acidic rain (pH = 5), the response times were extremely short and could not be accurately measured, as illustrated in Video S5 (Supporting Information), since the color change occurred instantaneously. The observed variation in response times between NH_3_ and pH sensors can be attributed to the distinct mechanisms of analyte interaction: ammonia vapors must diffuse through air into the porous structure and subsequently adsorb onto the sensor surface, whereas the absorption of acidic water occurs instantaneously due to the hydrophilic nature of the aerogel. Nonetheless, it is important to emphasize that, for sensors intended for field deployment with measurement intervals on the scale of hours or days, a response time of ≈3 min remains highly suitable and operationally efficient.

In Figure S9 (Supporting Information), we present a simulation of how a drone equipped with a camera at 4 m above the ground would capture the image of the pH sensor on the ground. The simulation was carried out, reducing the resolution of the image using ImageJ^[^ ^61^ ^]^ and accordingly with drone specifications reported by Bomantara et al.^[^ ^62^ ^]^ Interestingly, the drone would still be able to recognize a significant number of pixels (≈560) of the pH sensor, allowing for color analysis and, in the case of the pH sensor, accurate pH value measurement (estimated pH value was 5.1).

We later moved to demonstrate the two‐step degradation of our artificial samara, employing accelerated degradation conditions in Phosphate‐buffered Saline (PBS, pH = 7.40) at 37 °C (see Figure S8, Supporting Information for setup description). Degradation experiments were performed under static (no stirring, Figure 4c; Video S6, Supporting Information) and dynamic conditions (magnetic stirring, 400 rpm, Figure 4d; Video S7, Supporting Information). In both cases, the PVA wing dissolves first, particularly quickly when stirring was used (a few minutes), compared to the static case (≈3 h). Due to PVA's water‐solubility and the effect of moisture on its mechanical properties (in particular, on its stiffness), we believe that moist conditions, such as the ones found after moderate precipitations or after field irrigation, could be sufficient to start its degradation while fixing the sensor position. This is supported by the swelling and solubilization studies done by Symonds et al. on PVA films under lab and simulated rain conditions.^[^ ^50^ ^]^ Pleasingly, the CNCa sensors were completely disintegrated and degraded only after ≈5 h if dynamic conditions were employed, while no visible alteration was observed in the absence of stirring. The disintegration of CNCa sensors is due to the breakage of physical interaction between the CNC and the lack of chemical crosslinking. In the future, if a longer functional lifespan of the sensor is needed, chemical crosslinking can also be introduced to CNCa. These results show that the engineered design and fabrication principles applied to our artificial samara ensure a two‐step degradation of the entire unit, in accordance with sustainability guidelines in soft robotics, without compromising functionality.^[^ ^49^ ^]^

## 3 Conclusion

Based on the state of the art of eco‐friendly, bioinspired, wind‐dispersed fliers for environmental monitoring, we proposed an aerogel‐based colorimetric system inspired by the *T. tipu* samaras. Our design, materials choice, sensing principles, and fabrication strategy were driven by the limitations shown in past examples, our practical experience with image‐based environmental monitoring systems, as well as by sustainability and eco‐friendliness principles. The CNCa‐based colorimetric sensing flier presented here features a wide readout surface (≈3 cm^2^) for easy detection and exhibits strong adsorption of acidic rain and NH_3_, enabled by the high porosity of the sensor. Its sensing capabilities are endowed by safe, halochromic molecules extracted from edible sources such as red cabbage and turmeric. In a perspective scenario, wind will disperse the fliers, which are equipped with a water‐soluble wing in PVA designed to maximize dispersal distance through autorotation during descent. After landing, the wing will quickly degrade in wet conditions—such as moist soil after rainfall—leaving the sensor in a fixed position. In addition, the possibility of the development of a PVA blend with differing moisture resistance provides flexibility to optimize material choice depending on specific environmental conditions.^[^ ^63^ ^]^

The final design closely resembles the properties of the natural samaras, incorporating aerodynamic and fabrication considerations to ensure simplicity and functionality. To the best of our knowledge, this study is the first to quantify the morphometric and flight parameters of natural *T. tipu* samaras.

In perspective, the fliers will be deployed using drones that feature wireless control systems for environmental monitoring of remote, inaccessible, or polluted areas that are unsuitable for conventional environmental sampling and analysis systems. Moreover, given that our system measures pH and NH_3_, it is worth highlighting that these parameters are highly relevant not only for environmental monitoring in remote areas, but also for precision agriculture applications. After being released, they will be carried by the wind and eventually land on the ground (Figure S10, Supporting Information).^[^ ^64^ ^]^ The release will be in massive quantities (e.g., hundreds, redundancy) to compensate for inevitable losses or obscuration by foliage, and without causing environmental harm considering the (bio)degradability. Their final locations will then be identified using cameras installed on drones and machine learning and deep learning algorithms for the recognition and detection of seed‐like fliers by drones.^[^ ^62^ ^]^ The collected data will be retrieved using a camera equipped with RGB analysis systems (Figure S10, Supporting Information). The proposed bioinspired, responsive systems are designed for environmental signaling of acid rain events and monitoring the ammonia emissions from recently fertilized fields, both related to harmful and polluting species. The accuracy and precision of our sensors were evaluated in simulated environmental conditions and demonstrated reliable performance for the targeted applications. Moreover, to the best of our knowledge, this is the first report of a turmeric‐based sensor integrated into a deployable system for in situ monitoring of ammonia emissions in fertilized soils. The sensor's linear response range aligns with typical ammonia concentrations observed several hours after manure application, indicating its suitability for such monitoring tasks.^[^ ^59^ ^]^

This work lays the foundation for a new class of aerogel‐based sensors, which could, in principle, be extended to any type of colorimetric, fluorometric, or more broadly, optical sensors. Future efforts will focus on transitioning these systems from laboratory settings to real‐world environments, such as agricultural fields, grasslands, or even urban areas close to polluting sources, to further validate their performance. Owing to their versatility, these sensing units are well‐suited for incorporation into other soft robotic platforms for environmental monitoring. We envision their deployment through both passive (e.g., wind or water dispersal) and autonomous systems (e.g., energy‐harvesting or driven by stimuli‐responsive materials), including fliers, crawlers, and more complex mobile soft robots. The sensors’ lightweight nature, high sensitivity, and simple fabrication make them highly suitable for integration into these varied platforms.

## 4 Experimental Section

### Morphometric Analysis

Samaras of *T. tipu* were purchased on https://www.ebay.com from Bolivia. The same procedures were employed for both the natural samaras and the artificial fliers. Morphometric analysis was carried out using a digital caliper (RS PRO 150 mm Digital Caliper 0.0005 in, 0.01 mm, Metric & Imperial, UK) with a resolution of ± 0.01 mm and an optical microscope (KH‐8700, Hirox, Japan). The mass of the fliers was measured with an analytical balance (KERN ABS‐N, Germany) with a resolution of ± 0.0001 g. Wing surface (*S_w_*) was estimated from pictures of the fliers. By image processing with ImageJ,^[^ ^61^ ^]^ the flier capsule was deleted, and the resulting image was binarized. The wing surface was estimated by pixel counting, considering a scalebar of 2 cm. Some fliers (*n* = 3 for natural samaras, *n* = 5 for artificial samaras) were cut along the capsule border to calculate the *m_c_/m_w_* ratio.

### Aerodynamic Analysis

For descent speed (*v_d_*) measurements, the fliers were released from rest in a still air setting from a height of 3 m and allowed to fall freely. Tests were conducted indoors without active ventilation. The flight was recorded by a camera of a Samsung A13 (South Korea) or an Apple iPhone 15 (USA). The mean *v_d_* was calculated considering the time elapsed between the frame of the release and the frame in which the flier touched ground. The previously reported protocol was also applied for the measurement of the rotational angular velocity (*Ω*) and wing tip speed (*v_t_*). In this case, a smartphone camera (Apple iPhone 12 Pro Max (USA), 240 frames per second, 1280 × 800 pixels resolution for natural samples; Apple iPhone 15 (USA), 168 frames per second, 1920 × 1080 pixels resolution for artificial samples) was used to record video of a free fall. The previously reported protocol was also applied for the estimation of the conic angle *β*, determined by balancing of the centrifugal force acting on the distributed mass of the flier, the distributed weight of the flier, and the aerodynamic force that causes the driving moment of the flapping *x*‐axis.^[^ ^65^ ^]^ The angle variations were measured by the frames using ImageJ.^[^ ^61^ ^]^

### Design of the Artificial *T. tipu* Samara

A top‐view picture of a natural sample was taken using the camera (pixels) of a Samsung Galaxy A32 5G (South Korea) smartphone. A vector image file of the seed contour was obtained with Inkscape software, then it was imported into Siemens NX 11.0 CAD software and slightly adapted. The final flier design comprised a 0.10 mm‐thick wing and a circular capsule area (sensor base) with a diameter of 20 and a 1.00 mm thickness. Three 0.60 mm‐wide, 0.30 mm‐thick rectangular ribs were added onto the wing portion to reduce wing flexibility and improve the aerodynamic properties of the flier. The upper rib follows the flier curved profile, while the other two follow two straight lines that span across the wing portion. An .stl file was obtained and the corresponding .gcode file generated using PrusaSlicer software (slicing thickness 0.10 mm DETAIL at MK3, 100% infill, first layer height: 0.1 mm).

### 3D Printing of the Artificial *T. tipu* Samara

Using a PVA filament (Mowiflex 3D 2000, Kuraray, diameter: 1.75 mm) and a 0.4 mm nozzle, the water‐soluble carriers were printed on a Prusa i3 MK3S 3D printing machine (Prusa, Czech Republic), using the following conditions: nozzle temperature *T*_nozzle_ = 195 °C, printing bed temperature *T*_bed_ = 60 °C, printing speed *v* = 20 mm s^−1^ for first layer (wing), *v* = 40 mm s^−1^ for capsule.

### 10 wt.% CNC Hydrogel Preparation

Milli‐Q deionized water (22.5 g) was added to cellulose nanocrystals (CNCs, average width 10–20 nm, average length 300–900 nm, Nanografi Nanotechnology, 2.5 g) in a 50 mL beaker and stirred with an overhead stirrer (3000 rpm, FALC AT‐MD 20) until no solid particles were visible by eye. The hydrogel was transferred into a syringe barrel for DIW printing (30‐cc, Nordson EFD Optimum Syringe Barrel), capped (Nordson EFD Optimum series), and allowed to rest overnight.

### Aerogel Disk Design

The .stl file of a cylinder (*r* = 10 mm, *h* = 2.4 mm) was generated using Siemens NX 11.0 CAD software, sliced with Perfactory RP software (layer thickness: 0.500 mm), and the corresponding .bpl (Bioplotter file) was generated.

### Hydrogel Disk Printing

A hydrogel‐loaded syringe barrel was equipped with a 22GA‐nozzle (inner diameter: 0.41 mm/0.016″, SmoothFlow Tapered Dispense Tips, Nordson EFD). Aluminum foil was used as a printing base. The disks were printed with a DIW process, employing a 3D‐BIOPLOTTER (EnvisionTECH GmbH, Germany) 3D printing machine, controlled by the VisualMachines software. The following printing conditions were used: extrusion pressure *P* = 0.2 bar, printing speed *v* = 50 mm s^−1^, nozzle temperature *T*_nozzle_ = 20 °C, nozzle offset *z* = 0.500 mm, line pattern hatch type, distance between strands *d* = 0.40 mm, alternating layers with 90° and 0° orientation angles. When not used, the nozzle was kept moist by immersion of the tip in a 25 mL beaker full of Milli‐Q water. Nozzle purging was performed using a *P* = 0.4 bar after each layer.

### Aerogel Disks Making

A procedure based on solvent exchanges at room temperature and pressure conditions was adapted from the literature.^[^ ^54^ ^]^ Right after printing, the aluminum foil printing base was trimmed to fit inside a glass petri dish (120 mm diameter). The latter was capped and placed inside a freezer at −20 °C overnight to produce ice crystals within the gel structure. The next day, they were removed from the freezer and immediately fully submerged in ethanol (96.0–97.2%, Merck, Germany). They were allowed to thaw for 90 min to replace the ice crystals with ethanol while keeping the physical CNC network, before the ethanol was exchanged with acetone (≥99.5%, Merck, Germany). After 30 min, the acetone was removed and replaced with a fresh amount. A total of three, 30 minute‐long acetone baths were performed before allowing the disks to air dry at room temperature for ≈60 min.

### Scanning Electron Microscopy (SEM) Imaging

The gold‐sputtered (Quorum technologies Q150R‐ES, at 25 mA for 90s) surface of an aerogel disk was observed using a Zeiss EVO MA10 (*EHT*: 5.01 kV, *WD*: 8.41 mm, *I_probe_*: 65 mA).

### Assembly of the artificial *T. tipu* Samara

The PVA wing sensor base was wet with Milli‐Q water (1 drop), spread with a spatula, and then an aerogel sensor was gently pressed on it. The process was repeated on the other side of the carrier.

### Turmeric Dye Extraction

An existing procedure from Iqbal et al.,^[^ ^45^ ^]^ was adapted, where turmeric powder (40 g, store‐bought (Carrefour, Italy)) was extracted (50 °C, 400 rpm) in acetone (400 mL, Sigma–Aldrich, ≥99.5%) for 3.5 h in a capped 500 mL Erlenmeyer flask. The mixture was filtered on paper and the resulting liquid (360 mL of clear, orange solution) was stored at room temperature in 250 mL‐glass bottles, away from light, until further use.

### Red Cabbage Dye Extraction

A procedure from Kuswandi et al.^[^ ^44^ ^]^ was adapted. Red cabbage leaves (150 g, *Brassica oleracea var. capitata f. rubra*, store‐bought (CONAD SOCIETÀ COOPERATIVA, Italy)) were cut into pieces (≈1.5 × 3 cm in size), crushed in a mortar, then extracted at 50 °C in a 250‐mL Erlenmeyer flask containing ethanol (70 mL, 96.0–97.2%, Sigma–Aldrich) acidified with 25% HCl (300 µL, 25% HCl for analysis EMSURE, Merck Millipore) and occasionally stirred. After 180 min, the mixture was filtered using syringe filters (ReliaPrep 1.2 µm cellulose acetate syringe filters, Ahlstrom Munksjö). The filtrate (45 mL, clear, deep purple solution) had a pH = 4 (pH paper strips) and was collected in a 50 mL centrifuge test tube and stored at room temperature protected from light until further use. Just before aerogel dyeing, a known volume of the extract was neutralized using a 5 м NaOH aqueous solution, then diluted with ethanol (≥99.8%, Sigma–Aldrich) in a 1:1 volume ratio.

### Aerogel Disks Dyeing

An aerogel disk was placed on aluminum foil, and the extract (0.6–1.0 mL) was applied drop‐by‐drop on the upper surface of the disk until full percolation was achieved. The latter was visible due to the formation of a liquid meniscus at the bottom of the disk. The solvent was allowed to air dry (room temperature, 60 min) in the case of the NH_3(g)_ sensors, or in an oven (60 °C, 180 min) followed by 2 min in high vacuum (VP‐EC20‐1 Industrial Vacuum Pump (DVD Vacuum Technology, lowest achievable pressure: 2 mbar)) for pH sensors.

### NH_3_‐Responsive Sensor Testing and Calibration

NH_3_‐responsive sensors were placed on the base of in a PMMA rectangular box (purchased from https://www.amazon.com), together with an NH_3_ electrochemical sensor (operating range: 1–1000 ppm, FD‐NH3000 FORENSIC DETECTORS SD, USA). See Figure S3 (Supporting Information) for the setup. A 2.5% NH_4_OH solution (5 mL, prepared by dilution of a 28.0–30.0% (NH_3_ basis) ammonium hydroxide solution, ACS reagent, Sigma–Aldrich) was poured into a 5 mL glass beaker, which was used as a source of gaseous ammonia. The beaker was quickly placed on top of the box base, as close to the box border as possible. The box was immediately enclosed with the cover, and a video of the sensor chromatic response over time was recorded with a digital camera (NIKON D7500) on top of the enclosed chamber.

At different times, snapshots of the sensor were acquired, and their color (as RGB values) was analyzed using ImageJ software.^[^ ^61^ ^]^ The R, G, and B normalized values and the NH_3_ ppm value indicated on the electrochemical sensor were employed to build calibration curves. The same setup and procedure were employed on three sensors to test the accuracy and precision of the calibration curve.

### pH‐Responsive Sensor Testing and Calibration

Technical buffer solutions (Mettler Toledo), respectively of pH 4, 7, and 10 were applied drop‐by‐drop and until percolation ‐ over three different pH sensors. Top‐view pictures of each sensor were acquired with a digital camera (NIKON D7500) and their colour (in RGB mode values) evaluated using ImageJ software^[^ ^61^ ^]^ to build three different calibration curves (one for each normalized color channel). An acetate buffer solution (pH = 5, recorded using a SevenCompact S220 pH meter, Mettler Toledo) was prepared and applied to another three, anthocyanin‐dyed aerogel sensors to simulate an acid rain event and evaluate the performance of each calibration curve. The normalized R, G, and B were extrapolated using the same procedure described before for the turmeric sensors.

### Degradation Tests

Both static (no stirring) and dynamic (400 rpm) degradation tests were performed in PBS (500 mL, see Supporting Information for composition) at 37 °C, inside a 140 mm crystallizer. A top‐view video of the degradation setting was recorded using a webcam (Logitech Brio Ultra HD Pro Business Webcam) for 3 h. After 3 h, pictures were taken after 18 h (static conditions) or after 4 and 5 h (dynamic conditions) from the start, with a NIKON D7500 camera. See Figure S8 (Supporting Information) for the setup description.

### Statistical Analysis

Data are presented as mean ± SD, and error bars in the graph represent standard deviation (N samples range from 3 to 24).

## Conflict of Interest

The authors declare no conflict of interest.

## Author Contributions

G.G. contributed to conceptualization, performed the experiments, wrote, edited, and reviewed the manuscript; R.T. contributed to conceptualization and reviewed the manuscript. C.F. performed the aerogel structural investigations with SEM. S.M. contributed to conceptualization, performed the flight experiments on natural *Tipuana tipu* samples, provided advisory suggestions, supervised and validated experiments, and contributed to the writing and review of the manuscript. B.M. contributed to conceptualization, directed the research, funding, and revised the manuscript.

## Supporting information



Supporting Information

Supplemental Video 1

Supplemental Video 2

Supplemental Video 3

Supplemental Video 4

Supplemental Video 5

Supplemental Video 6

Supplemental Video 7

## Data Availability

The data that support the findings of this study are available from the corresponding author upon reasonable request.
