# Supplementary material for: A Degradable Bioinspired Flier with Aerogel‐Based Colorimetric Sensors for Environmental Monitoring
Source: Adv Sci (Weinh). 2025 Aug 28;13(15):e08949. doi: 10.1002/advs.202508949 (PMC13042518; doi:10.1002/advs.202508949)
Supplement: Supplementary file 1 — Supporting Information [file ADVS-13-e08949-s004.docx]

Supporting Information

**A Degradable Bioinspired Flier with Aerogel-Based Colorimetric Sensors for Environmental Monitoring**

*Gianpaolo Gallo,^1^ Ruowen Tu,^1^ Carlo Filippeschi,^1^ Stefano Mariani,^1*^ and Barbara Mazzolai^1*^*

^1^Bioinspired Soft Robotics Laboratory Istituto Italiano di Tecnologia, Via Morego 30, Genova 16163, Italy

*E-mail: stefano.marian.iit@gmail.com; barbara.mazzolai@iit.it


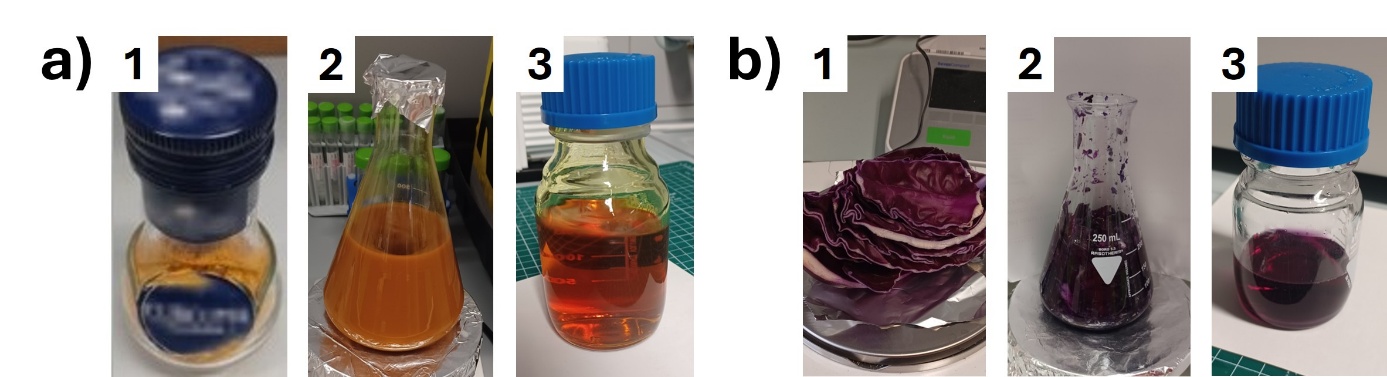


**Figure S1.** a) turmeric and b) red cabbage extraction steps (raw material, extraction, dyeing solution).


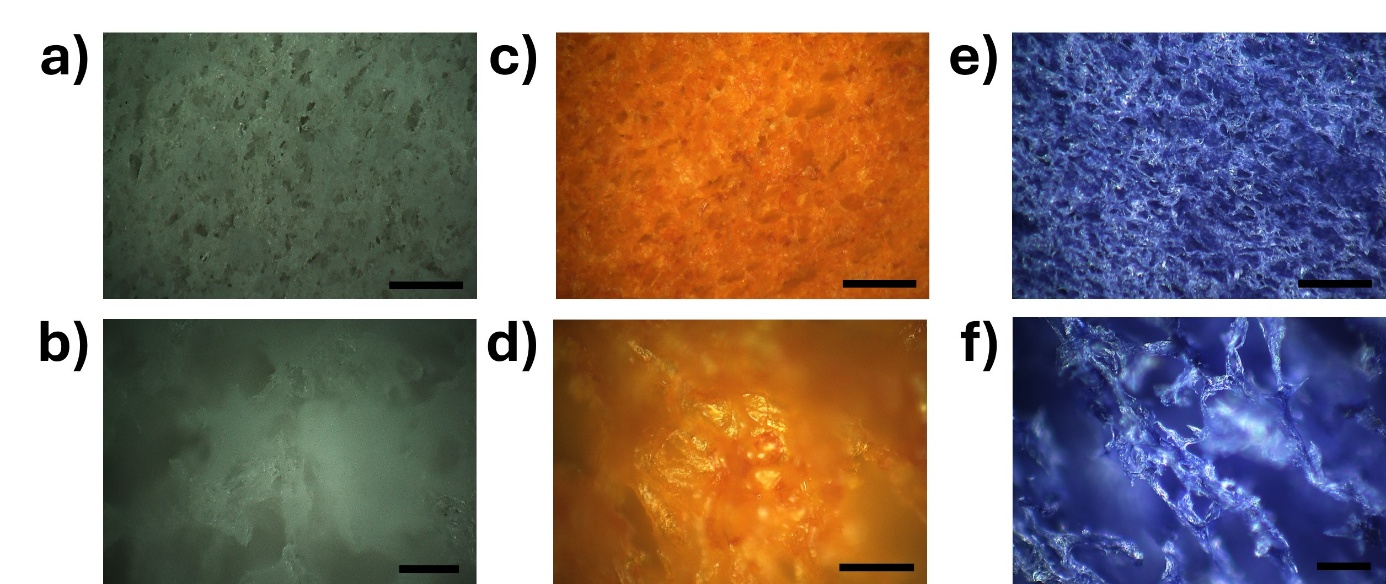


**Figure S2.** Optical microscope pictures of the porous aerogel structure: a-b) before dyeing, c-d) after dyeing with turmeric extract, e-f) after dyeing with red cabbage extract. Scalebars are 500 μm for a),c) and e) and 100 μm for b),d) and f).


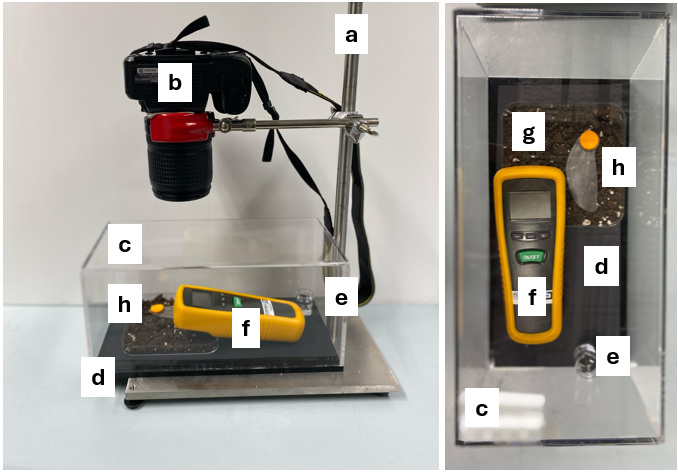


**Figure S3.** Lateral (left) and top-view (right) pictures of the setup used to record the colorimetric response of turmeric-based sensors to gaseous ammonia. a) stand, b) camera c) box transparent enclosure, d) box base, e) NH_3(g)_ source, f) Electronic NH_3(g)_ sensor, g) Petri dish with topsoil (video background), h) artificial *Tipuana* with colorimetric, turmeric-based sensor.


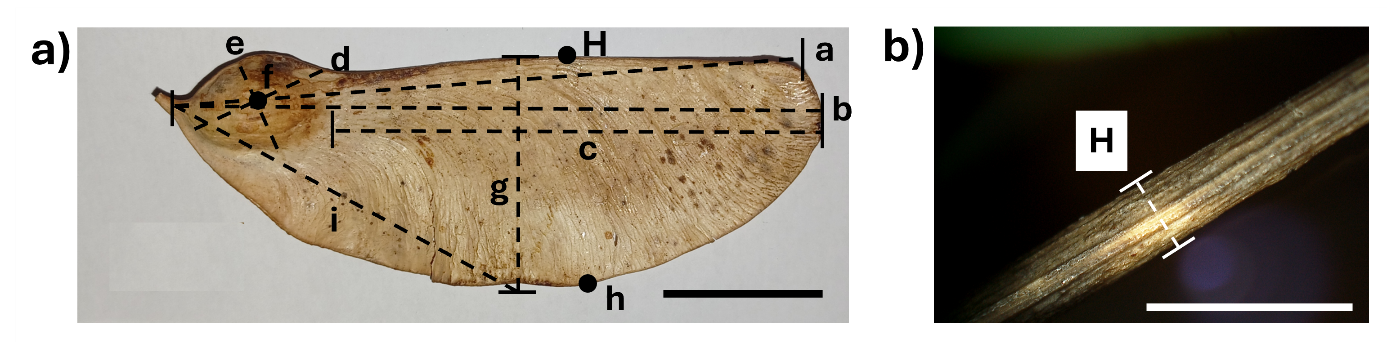


**Figure S4.** Picture of *Tipuana* *tipu* seed and morphometric parameters where: *a* is tip-to-corner length; *b* is tip-to-wing tail length; *c* is capsule-to-wing tail length; *d* is capsule major axis; *e* is capsule minor axis; *f* is capsule thickness; *g* is the wing chord; *h* is the lower rib thickness; *H* is major rib thickness; *i* is tip-to-chord length. Scalebar is 2 cm. b) Picture of the major wing thickness chord. Scalebar is 1 mm.

**Table S1.** Morphometric measurements of natural *T. tipu* samaras. Sample pool *n* = 6; for *h* and *H*, at least 6 measurements (on the same samara but in different points) were taken and used to calculate the average value for an average samara.

| Length code^a)^ | Mean value  [mm] | Standard Deviation  [mm] |
| --- | --- | --- |
| *a* | 76 | 5 |
| *b* | 77 | 4 |
| *c* | 59 | 3 |
| *d* | 18 | 2 |
| ***e*** | 12 | 1 |
| *f* | 8.3 | 0.4 |
| *g* | 31 | 2 |
| *h* | 0.094 | 0.022 |
| *H* | 0.260 | 0.014 |
| *i* | 47 | 3 |

^a)^ See Figure S4 for lengths specification.

**Table S2.** Measurements of relevant parameters for evaluation of aerodynamic properties of natural *T. tipu* samaras. Error expressed as standard deviation over a sample pool size of *n* = 7 for *m*, *S_w_* and *W*/*S_w_*, *n* = 3 for *m_c_/m_w_* and *n* = 6 for the others.

| **Total mass**  ***m* [g]** | **Capsule-to-wing ratio**  ***m_c_/m_w_*** | **Wing surface**  ***S_w_* [cm^2^]** | **Wing loading *W/S_w_***  **[N m^-2^]** | **Descent speed**  ***v_d_* [m s^-1^]** | **Rotational velocity**  ***Ω* [rad s^-1^]** | **Wing tip speed**  ***v_t_* [m s^-1^]** | **Coning angle**  ***β* [°]** | **Reynolds number *Re*** | **Descent Factor**  ***DF*** |
| --- | --- | --- | --- | --- | --- | --- | --- | --- | --- |
| 0.9 ± 0.2 | 3.9 ± 0.8 | 17.2 ± 1.7 | 4.9 ± 0.7 | 1.4 ± 0.2 | 57.9 ± 8.3 | 4.4 ± 0.7 | 26.3 ± 0.8 | 2978 ± 395 | 2.0 ± 0.3 |


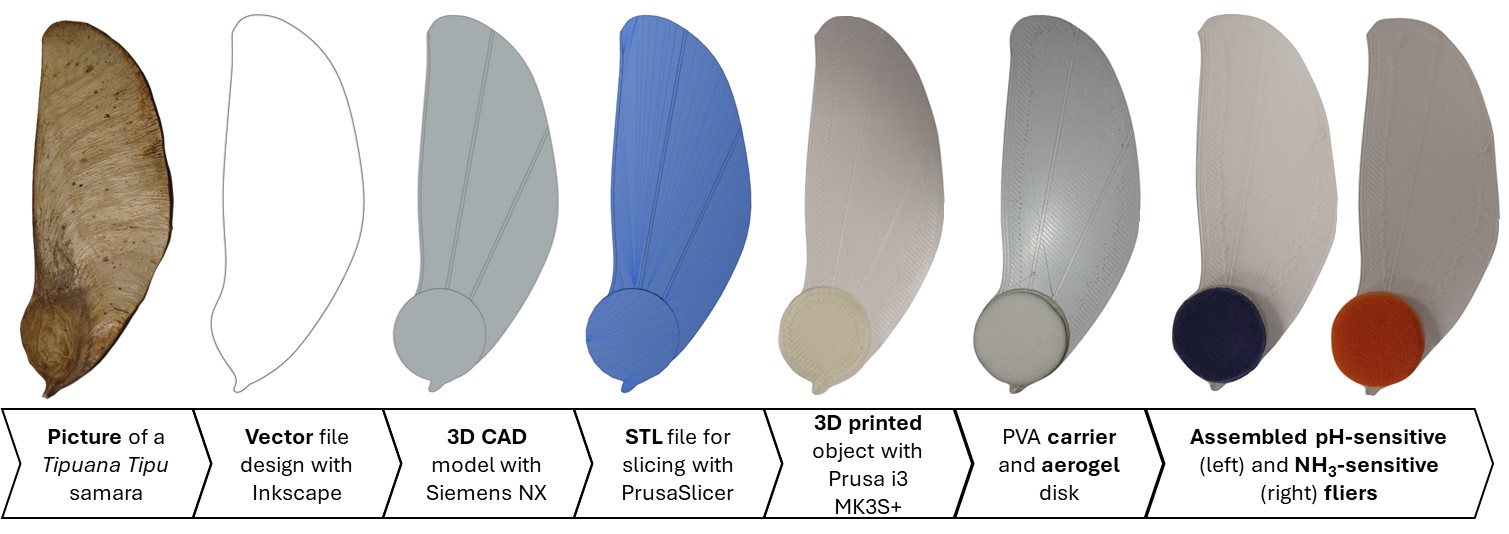


**Figure S5.** Flow chart for the 3D printing of the artificial *Tipuana* consisting of: (i) picture of a *Tipuana tipu* seed; (ii) drawing of the contours and creation of a vector file; (iii) creation of a 3D CAD model; iv) creation of a .stl file; (v) 3D printing of the artificial; (vi) carrier and aerogel capsule, (vii) loading of sensitive and biodegradable molecules in the aerogel.

**Table S3.** Comparison between natural and artificial *Tipuana* features. *Sample pool size: *n =* 8 for *S_w_*; *n =* 7 for *Ω*, *v_t_* and *β; n =* 5 for *m_c_/m_w_*; *n* = 6 for the others.

|  | Total mass *m* [g] | Capsule-to-wing mass ratio *m_c_/m_w_* | Wing surface *S_w_*  [cm^2^] | Wing loading *W/S_w_*  [N m-^2^] | Descent speed *v_d_* [m s^-1^] | Rotational velocity *Ω* [rad s^-1^] | Wing tip speed *v_t_* [m s^-1^] | Coning angle  *β*  [°] | Reynolds number *Re* | Descent Factor *DF* |
| --- | --- | --- | --- | --- | --- | --- | --- | --- | --- | --- |
| Natural *T.Tipu* | 0.9 ± 0.2 | 3.9 ± 0.8 | 17.2 ± 1.7 | 4.9 ± 0.7 | 1.4 ± 0.2 | 57.9 ± 8.3 | 4.4 ± 0.7 | 26.3 ± 3.8 | 2978 ± 395 | 2.0 ± 0.3 |
| Artificial *T.Tipu* *** | 0.91 ± 0.04 | 3.3 ± 0.2 | 15.9 ± 0.5 | 5.6 ± 0.4 | 1.8 ± 0.3 | 68.2 ± 3.3 | 5.6 ± 0.3 | 24.2 ± 1.7 | 3635 ± 688 | 1.7 ± 0.3 |


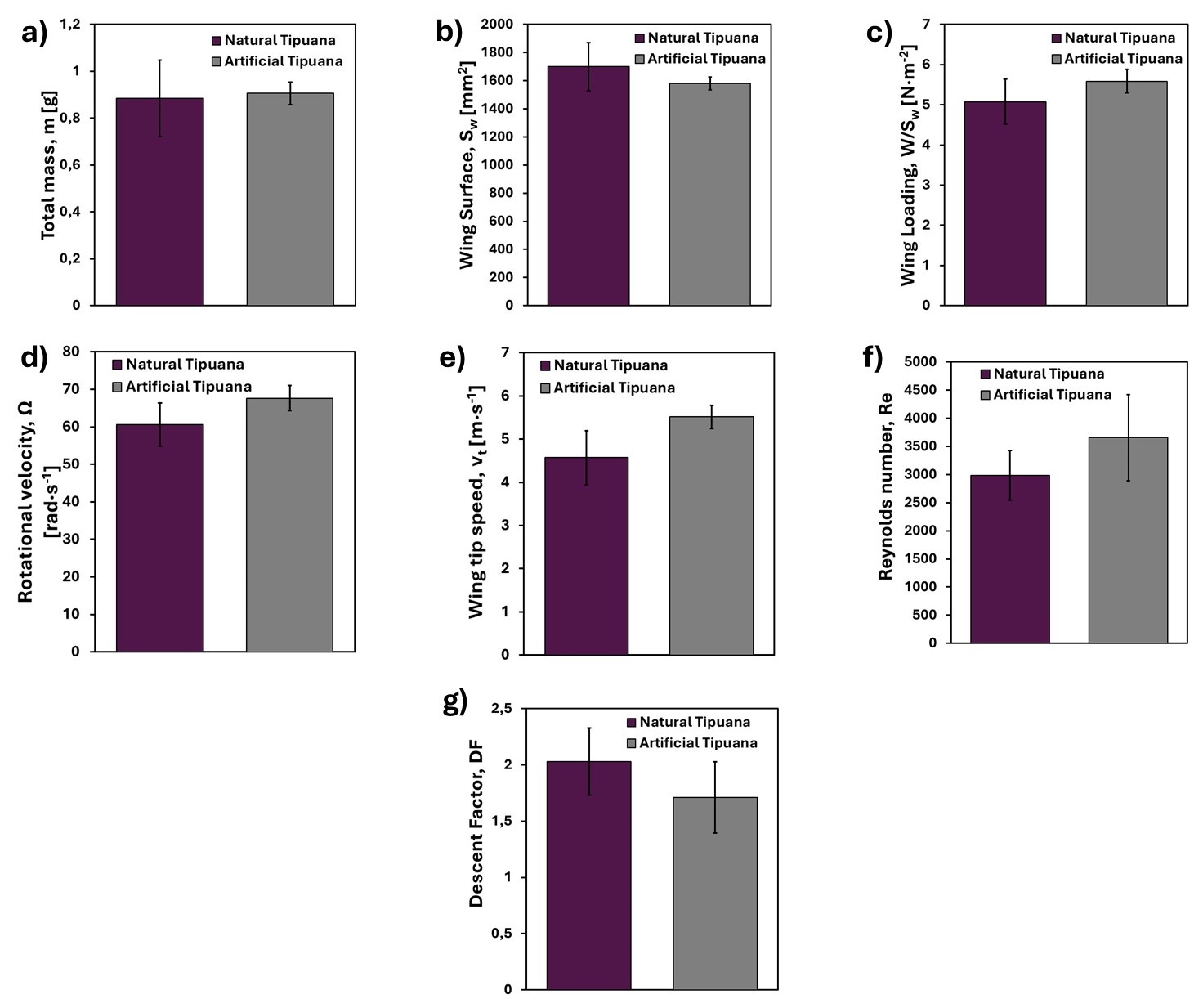


**Figure S6.** Natural (purple) and artificial (gray) *Tipuana* comparative graphs for a) total mass *m*, b) wing surface *S_w_*, c) wing loading *W/S_w_*, d) rotational velocity *Ω*, e) wing tip speed *v_t_*, f) Reynolds number *Re*, g) descent factor *DF*. The quantities shown are obtained from Table S3 and represented as mean ± standard deviation.

**PBS preparation for degradability tests**

Phosphate Saline Buffer (PBS) was prepared mixing NaCl (16.0146 g, 274.0 mol, ≥99.5%, Sigma Aldrich), KCl (0.4047 g, 5.4 mmol, ≥99.0%, Sigma Aldrich), Na_2_HPO_4_·2H_2_O (3.5598 g, 20.0 mmol, ≥98.0%, Sigma Aldrich), KH_2_PO_4_ (0.4917 g, 3.6 mmol, ≥99.0 %, Sigma Aldrich) and Milli-Q water (2 L). After complete dissolution of all the solids, a pH of 7.40 was measured (SevenCompact S220 pH meter, Mettler Toledo).


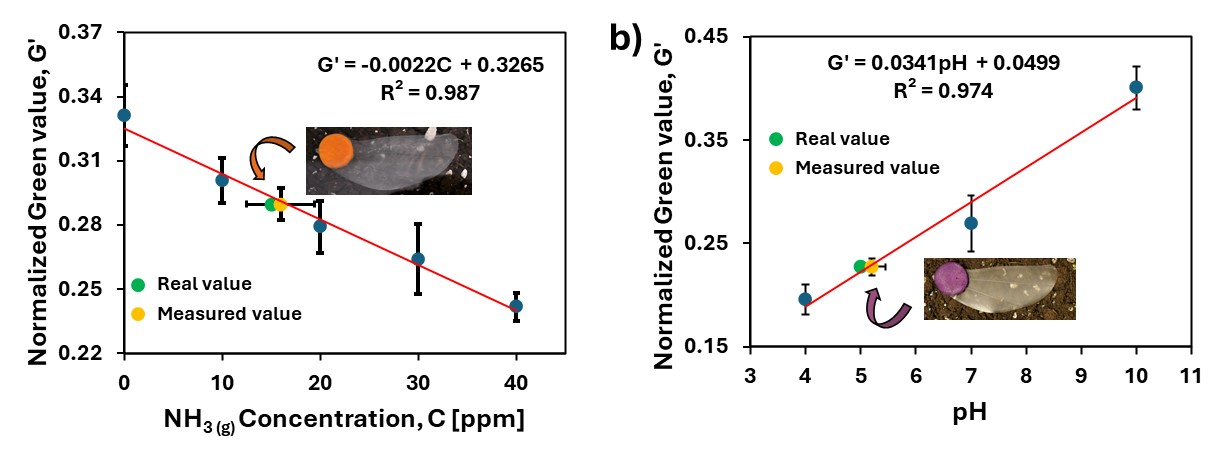


**Figure S7.** Sensing in simulated environments. a) Calibration curve for NH_3_(g) sensors and response of artificial *Tipuana* fliers integrated with NH_3(g)_ sensors at C = 15 ppm (the green bullet represents the real value while the yellow bullet represents the measured value using RGB analysis). b) Calibration curve for pH sensors and response of artificial *Tipuana* flier integrated with pH sensors at pH = 5 (green bullet represents the real value while the yellow bullet represents the measured value using RGB analysis). Data are presented as mean ± SD, and error bars indicate standard deviations (N samples = 3-5).


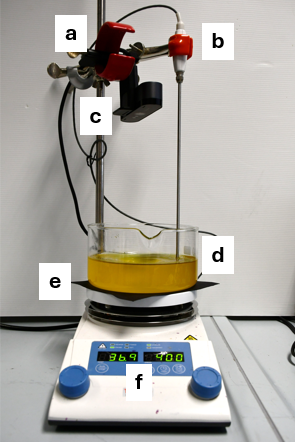


**Figure S8.** Degradation setup, lateral view. a) stand, b) temperature probe, c) webcam, d) degradation vessel, e) black cardboard (video background), f) hotplate.


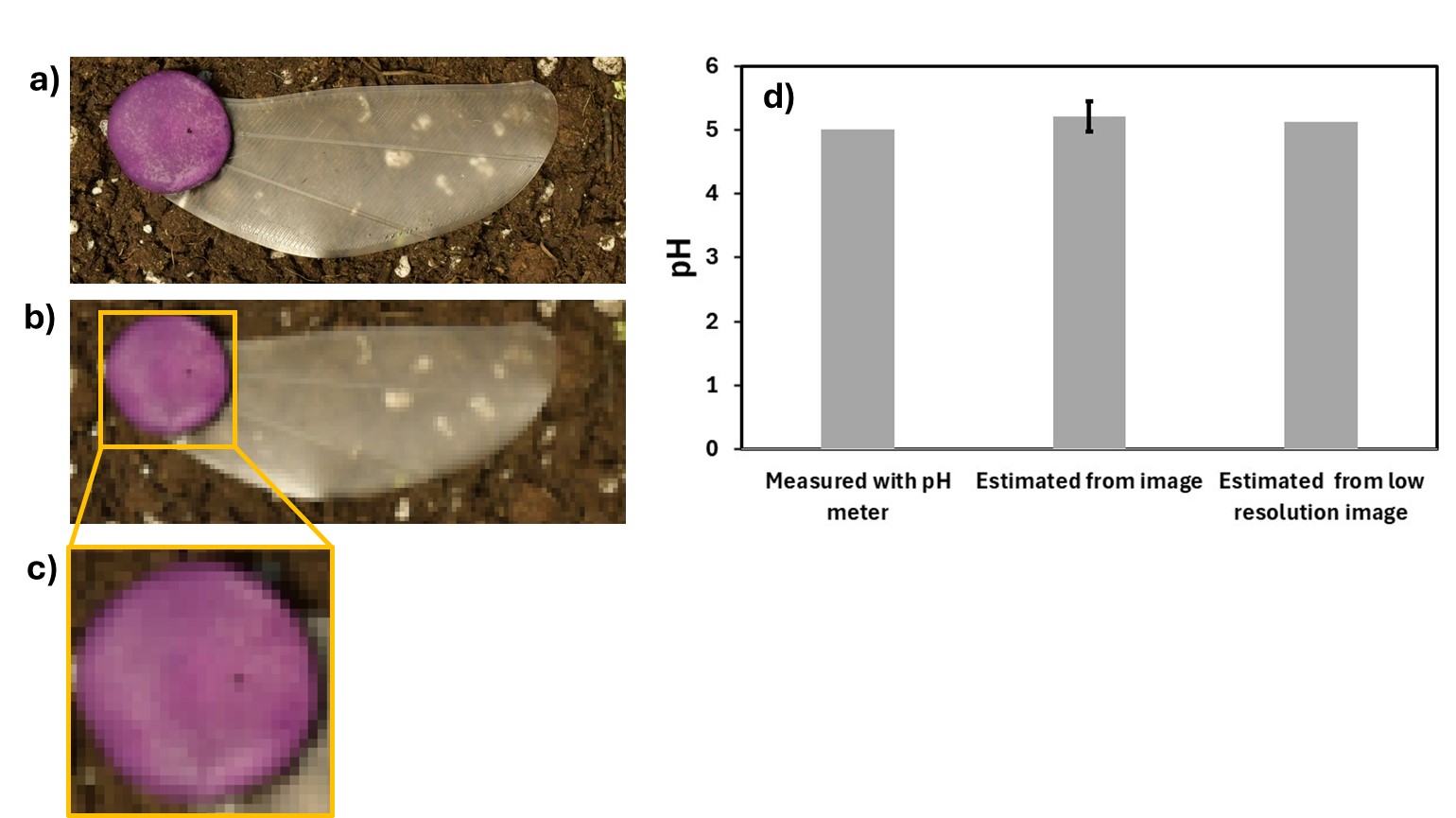


**Figure S9.** Simulation of drone-based detection of pH-responsive sensors. a) Laboratory image of the pH-sensitive *samara* before and after exposure to simulated acid rain (pH = 5 acetate buffer), captured using a Samsung Galaxy A32 5G (South Korea) at a resolution of 720 × 1600 pixels. b) Simulated drone view of a ground-deployed pH sensor taken from an altitude of 4 meters. Image resolution was downscaled in ImageJ^[1]^ according to the specifications of Bomantara et al.^[2]^ yielding an estimated spatial resolution of approximately 0.75 mm/px. c) Enlarged view of the pH sensor shown in panel (b). d) Comparison of pH values obtained by direct measurement with a pH meter, by image analysis under laboratory conditions, and by estimation from the low-resolution simulated drone image, based on the method described by Bomantara et al.^[2]^

**
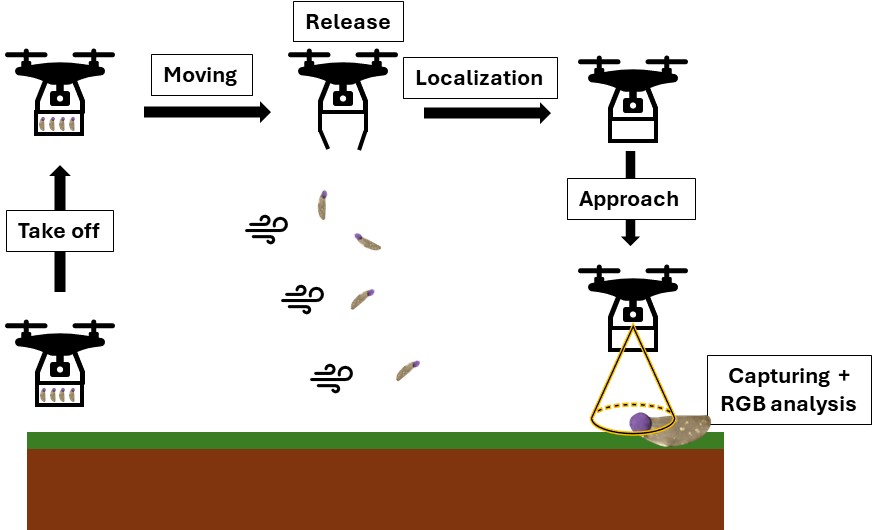
**

**Figure S10.** Strategy of using seed-like fliers integrated with CNC colorimetric sensor. The strategy consist of: loading of fliers into a wireless controlled releaser coupled with the drone; take off; flying and moving in the air before deploying the fliers; release of the fliers carried away from the wind; geolocalization of the fliers with the drones using a camera and machine learning and deep learning algorithms;^[2]^ readout of the sensors on the topsoil using drones equipped with RGB camera technology. Reproduced from Ref. A. Nexha, S. Mariani, K. Cikalleshi, T. Kister, B. Mazzolai, T. Kraus, Nanoscale 2025.with permission from the Royal Society of Chemistry, under a Creative Commons Attribution 3.0 Unported Licence.^[3]^

**References**

1. C. A. Schneider, W. S. Rasband, K. W. Eliceiri, *Nat. Methods* **2012**, *9*, 671.
2. Y. A. Bomantara, H. Mustafa, H. Bartholomeus, L. Kooistra, *Remote Sensing*, **2023**, *15(6)*, 1637.
3. A. Nexha, S. Mariani, K. Cikalleshi, T. Kister, B. Mazzolai, T. Kraus, *Nanoscale* **2025**. In press. DOI: 10.1039/D5NR01318F.

**VIDEO**

**Video S1.** Lateral view of the descent of a natural *Tipuana Tipu* samara, launched from a height of about 3 m, showing spontaneous autorotation.

**Video S2.** Bottom view of the descent of a natural *Tipuana Tipu* samara, launched from about a height of about 3 m, showing spontaneous autorotation.

**Video S3.** Lateral view of the descent of an artificial *Tipuana*, launched from a height of about 3 m, showing spontaneous autorotation.

**Video S4.** Bottom view of the descent of an artificial *Tipuana*, launched from about a height of about 3 m, showing spontaneous autorotation.

**Video S5.**  Response of colorimetric sensors for pH and NH_3_.

**Video S6.** Static degradation of an artificial *Tipuana* in PBS (pH 7.40, 500 mL) at 37°C over the first 3h.

**Video S7.** Dynamic (400 rpm) degradation of an artificial *Tipuana* in PBS (pH = 7.40, 500 mL) at 37°C over the first 3h.
